# Clinical Characteristics and Dental Treatment of Patients Attending a UK Dental Hospital—A Descriptive Study

**DOI:** 10.1002/cre2.70427

**Published:** 2026-08-10

**Authors:** Jing Kang, Chenyi Gao, Nikolaos Donos, Vanessa Muirhead, Sue Pavitt, Jianhua Wu

**Affiliations:** ^1^ Centre of Clinical Translational Science, Faculty of Dentistry, Oral, and Craniofacial Sciences King's College London London United Kingdom; ^2^ School of Dentistry University of Leeds Leeds United Kingdom; ^3^ Wolfson Institute of Population Health Queen Mary University of London London United Kingdom; ^4^ Centre for Oral Clinical Research, Institute of Dentistry Queen Mary University of London London United Kingdom

**Keywords:** big data, descriptive study, electronic health records, epidemiology, oral health

## Abstract

**Objectives:**

To describe the clinical characteristics and dental treatment for patients treated at a dental hospital in the United Kingdom.

**Material and Methods:**

This descriptive study utilized anonymized routinely collected electronic health record data from dental patients who attended Leeds Dental Institute, collected from 2014 to 2023. Patient characteristics, comorbidities and completion of dental treatment were reported through descriptive statistics (mean and standard deviation [SD]; percentage) and by clinic attended.

**Results:**

A total of 109,718 patients were included in this study, of whom 58,964 (54%) were female, and 43,828 (40%) lived in the most deprived areas in England. The most frequently recorded comorbidities were joint or bone problems (*n* = 4913, 5%), chest or breathing problems (*n* = 4619, 4%), and heart problems (*n* = 4455, 4%). Across clinic groups, the proportion of patients with at least one recorded comorbidity was highest among those attending periodontal/restorative clinics (24%) and acute dental care clinics (19%). Less than half of all patients (*n* = 52,655, 48%) completed their dental treatment. Oral surgery had the highest percentage of patients who completed their treatment (*n* = 10,459, 71%).

**Conclusions:**

The sociodemographic and clinical characteristics reported in this study reflect a large population of dental patients from Leeds and the surrounding region. The findings of this study are consistent with literature showing sociodemographic trends in dental attendance. Future linkage of dental electronic health records with hospital records could improve understanding of the complexities of oral‐systemic associations further.

## 1 Introduction

The scarcity of research‐ready dental data (both within the United Kingdom and globally) presents a significant barrier to advancing understanding of oral‐systemic disease links and associations. This issue is a particular challenge in the United Kingdom because of the dearth of databases incorporating both dental and medical information from representative populations; this hampers observational research. While routinely collected medical datasets in primary and secondary care could be an invaluable resource for medical research (Herrett et al. 2015), dental research is severely limited by fragmented and non‐standardized reporting, privatization of data, and in some cases, continued reliance on paper records.

The United States and South Korea have developed nationally representative datasets that include comprehensive clinical dental and medical information. For instance, the National Health and Nutrition Examination Survey (NHANES) in the United States and the Korea National Health and Nutrition Examination Survey provide extensive data on clinical oral health, comorbidities, and lifestyle, which facilitate valuable insights into epidemiological associations within dentistry (Oh et al. 2021; Centers for Disease Control and Prevention CDC 2024). Furthermore, US researchers have successfully developed a research‐ready, nationally representative dental electronic health records (dEHR) dataset (Thyvalikakath et al. 2020), underscoring the feasibility and benefits of such resources. However, extrapolating findings from these resources to the United Kingdom context is problematic because of the sociodemographic and cultural differences across nations.

The UK Adult and Children Dental Surveys are conducted every 10 years to study the trends in the prevalence and determinants of oral diseases in England, Wales, and Northern Ireland. The surveys also collect information on dental attendance, clinical oral measurements, diet, as well as a self‐rated general health indicator (Chenery 2011). The long time period between this repeated cross‐sectional survey and the limited collection of data about other chronic diseases and comorbidities has hindered a robust data analysis to assess the links between oral‐systemic diseases. Previous research using Big Data has assessed associations between oral and general systemic health in the United Kingdom (Yi et al. 2023; Larvin et al. 2020, 2021; Kang et al. 2023). However, findings from these studies are limited to self‐reported oral health indicators rather than clinical data to accurately define oral diseases. Other epidemiological studies have used surveys to explore oral health and quality of life (Tsakos et al. 2012). Sociodemographic data collected in dEHR from patients who attend primary care dental practices have also been explored (West et al. 2020). Recently, a single‐centre study of dental patients attending a tertiary clinic in Birmingham showed the advantages of using dEHR in research (Gadd et al. 2024). Despite the relatively small sample size (*n* = 166), the study showed that dEHR can be successfully used as a data resource in the United Kingdom. Developing a large‐scale, research‐ready dental dataset that capitalizes on the availability of routinely collected electronic records in the healthcare system will facilitate and advance dental research in the United Kingdom.

In the United Kingdom, dental hospitals provide specialist oral healthcare beyond the scope of general dental practice, serving as tertiary referral centers for patients with complex diagnoses, high procedural risk, or requirements for specialist facilities and complex surgery. Consequently, dental hospital populations are typically more clinically complex and heterogeneous than those seen in primary dental care, encompassing patients across wide age and socioeconomic ranges, including those with multimorbidity, polypharmacy, disability, frailty, or rare and diagnostically challenging conditions.

This study reports on the first established research‐ready dental dataset using routinely collected dEHR for patients who attended a dental hospital in Leeds for treatment. It describes the patient characteristics and quantifies the patient population who completed their dental treatment to demonstrate the utility and value of this unique research‐ready dataset.

## 2 Materials and Methods

### 2.1 Design

This is a descriptive study utilizing the dEHRs of all patients who attended the Leeds Dental Institute (LDI) between 2014 and 2023. The index date of this study was the date of first recorded attendance at LDI. Dental EHR from 2014 onwards were included because the LDI introduced SALUD, a computer‐based electronic patient record and administration system, in 2014. Before its implementation, patient records were not routinely captured in an electronic format.

### 2.2 Study Population

**Figure 1:**
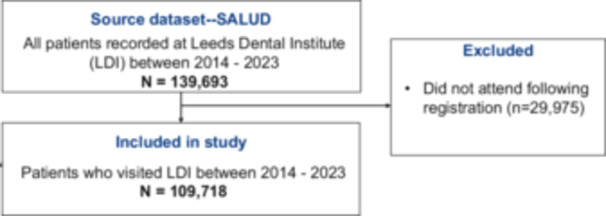
Flow diagram of this descriptive study.

The participants included in this study were those who attended LDI for any problems and treatments. Patients who were referred but did not ever attend their appointments at LDI were excluded. A simple flow diagram is presented to demonstrate the study design (Figure 1).

### 2.3 Setting

Patient dEHR were extracted from the Salud reporting database at LDI. SALUD is an academic dental software that allows recording collection of dEHR (Salud—academic dental software Internet 2024). LDI is a National Health Service (NHS) secondary dental care provider within the Leeds Teaching Hospitals NHS Trust (LTHT) in partnership with the University of Leeds and complies with the NHS National Data Opt‐Out scheme, whereby patients may choose to remove their consent for their data to be used for research. Notices on how data are used are available for patients to review in waiting rooms at the dental hospital. LDI serves an ethnically and economically diverse population living primarily in Leeds and West Yorkshire, with approximately 110,000 patients seen annually and 60,000 new referrals. LDI was a relatively early adopter of dEHR.

The LDI dEHR comprises information on patient demographics as well as administrative and clinical information. Patients who attend LDI are recorded using their NHS number as a unique identifier, which also minimizes the potential for duplicated records within the data. All study data were retrospectively extracted and de‐identified by the Research Data and Informatics Team at LTHT. NHS numbers were excluded from the dataset, and all patients were assigned pseudonymized ID numbers before data delivery.

### 2.4 Ethical Approval

This study received full NHS Health Research Authority approval (IRAS reference: 277767).

### 2.5 Treatment Status and Clinic Types

Treatment completion was defined using two fields in the dental electronic health record: appointment status and appointment outcome. Appointment status is recorded in the description variable and included the following categories: “Attended,” “Booked,” “Canceled,” “Did Not Attend (DNA),” “Pre Salud,” and “Rescheduled.” Appointment outcome was recorded in the appointment record note and included the following categories: “Discharged,” “Discharged – No Tx provided,” “Discharged – Tx Complete,” “Follow Up Patient – Select outcome below,” “Further appointment required,” “New Patient – Started Watchful Waiting,” “New Patient – Treatment not Started,” “New Patient – Treatment Started Today (TA),” “Patient DNA – Discharged (DP),” “Patient DNA – Ortho Hold for 1 Month (DA),” “Patient DNA – Reappoint – only use in exceptional circumstances (DA),” or “Sent to LGI on the day.” For this study, treatment completion was defined as “Yes” only for appointments with a status of “Attended” and an appointment outcome of “Discharged – Tx Complete.” Attended appointments with other recorded outcomes were classified as not completed.

Clinic type was derived from the clinic description variable. Eleven clinic types were identified: acute dental care, maxillofacial surgery, oral medicine, oral surgery, orthodontics, periodontal/restorative, pediatrics, sedation, hygiene and therapy, prosthetics, and Other. Six clinics were the focus of this study, including acute dental care, maxillofacial surgery, oral medicine, oral surgery, orthodontics, and periodontal/restorative. The remaining five clinic groups were presented in the supplement because of their small sample size or data accuracy concerns (e.g., pediatric clinic records had patients aged more than 20 years; Table S1).

### 2.6 Other Variables

Age was reported in 10‐year age bands; patients aged 14 years and below were denoted as “≤ 14 years” and those older than 64 as “≥ 65 years.” Patients aged 14 years or less were defined as pediatric patients. Sex was male, female, and unknown. Index of Multiple Deprivation (IMD) was quantified using the mean index value according to partial patient postcode, reported as quintiles (1 as most deprived and 5 as least deprived).

The dataset also comprised self‐reported comorbidities. Patients were asked: “Do you have X?,” where X was a condition defined as blood‐borne diseases, bruising/bleeding disorders, cancer, chest or breathing problems, diabetes, epilepsy or seizures, heart problems, joint or bone problems, liver disorders or kidney disease, or mental health illness. No clinically validated diagnoses or diagnostic codes were associated with these responses. Pregnancy or breastfeeding was also self‐reported. Clinical information also available in Salud included periodontitis charting and OPCS‐4 codes for dental treatment, but it was beyond the scope of the present study; therefore, it was not reported.

The time‐related variables were calculated and derived from the duration from the date of registration (set as day 0) and the number of days between the date of registration at LDI and the last appointment before hospital discharge.

### 2.7 Statistical Analysis

Patient characteristics were descriptively explored. Demographics are presented as means with standard deviations (SD), or medians with interquartile ranges (IQR) for continuous variables depending on the distribution of data; frequency (*n*) and percentage (%) are also reported for categorical variables. Patient information including age, deprivation and self‐reported comorbidities were studied according to the clinic the patient first attended. We also report the average number of appointments attended per patient and the proportion of patients with completed treatment. Missing data are reported within tables. Results are tabulated and visualized using bar plots and heat maps.

All analyses and data processing were conducted in R version 4.3.0. This report conforms to STROBE and RECORD guidelines.

## 3 Results

### 3.1 Summary of Study Population

Overall, there were 139,693 patients registered at LDI during the period from 2014 to 2023. Following exclusion of patients who did not attend the dental hospital following referral (*n*: 29,975 patients, Figure 1), there were 109,718 unique patients included in this study. Almost one quarter of first recorded attendances at LDI were in periodontal/restorative clinics (*n*: 24,394 patients, 22.2%; Table 1).

**Table 1:** Patient characteristics of the total study population and by first clinic attended.

		Clinic					
	Study population	Acute dental care	Maxillofacial surgery	Oral medicine	Oral surgery	Orthodontics	Periodontal/restorative
Total, *N*	109,718	10,447	15,426	10,539	14,702	6364	24,394
Age in years, *N* (%)							
≤ 14	21,748 (19.8%)	32 (0.3%)	851 (5.5%)	463 (4.4%)	1095 (7.4%)	3334 (52.4%)	143 (0.6%)
15–24	13,706 (12.5%)	1833 (17.5%)	1911 (12.4%)	562 (5.3%)	1933 (13.1%)	2104 (33.1%)	2788 (11.4%)
25–34	15,391 (14.0%)	2394 (22.9%)	2608 (16.9%)	1036 (9.8%)	2669 (18.2%)	495 (7.8%)	3702 (15.2%)
35–44	13,914 (12.7%)	1850 (17.7%)	2258 (14.6%)	1471 (14.0%)	2156 (14.7%)	231 (3.6%)	4135 (17.0%)
45–54	15,026 (13.7%)	1721 (16.5%)	2356 (15.3%)	1916 (18.2%)	2106 (14.3%)	126 (2.0%)	5100 (20.9%)
55–64	13,403 (12.2%)	1278 (12.2%)	2148 (13.9%)	2171 (20.6%)	2019 (13.7%)	49 (0.8%)	4246 (17.4%)
≥ 65	16,530 (15.1%)	1339 (12.8%)	3294 (21.4%)	2920 (27.7%)	2724 (18.5%)	25 (0.4%)	4280 (17.5%)
Sex, *N* (%)							
Female	58,964 (53.7%)	4280 (41.0%)	8039 (52.1%)	6712 (63.7%)	8595 (58.5%)	3537 (55.6%)	13,202 (54.1%)
Male	50,742 (46.2%)	6166 (59.0%)	7385 (47.9%)	3826 (36.3%)	6107 (41.5%)	2826 (44.4%)	11,189 (45.9%)
Missing	12 (0.0%)	1 (0.0%)	2 (0.0%)	1 (0.0%)	0	1 (0.0%)	3 (0.0%)
IMD quintile, *N* (%)							
1 (most deprived)	43,828 (39.9%)	5029 (48.1%)	6190 (40.1%)	3531 (33.5%)	5743 (39.1%)	2574 (40.4%)	8596 (35.2%)
2	22,481 (20.5%)	2335 (22.4%)	3016 (19.6%)	1982 (18.8%)	2678 (18.2%)	1316 (20.7%)	5768 (23.6%)
3	16,754 (15.3%)	1313 (12.6%)	2599 (16.8%)	1765 (16.7%)	2455 (16.7%)	1001 (15.7%)	3460 (14.2%)
4	17,419 (15.9%)	1225 (11.7%)	2463 (16.0%)	2183 (20.7%)	2620 (17.8%)	985 (15.5%)	3947 (16.2%)
5 (least deprived)	9045 (8.2%)	506 (4.8%)	1137 (7.4%)	1058 (10.0%)	1183 (8.0%)	476 (7.5%)	2588 (10.6%)
Missing	191 (0.2%)	39 (0.4%)	21 (0.1%)	20 (0.2%)	23 (0.2%)	12 (0.2%)	35 (0.1%)
Mean registration period, years (SD)	1.1 (1.7)	0.7 (1.5)	0.8 (1.4)	1.0 (1.6)	0.8 (1.3)	2.2 (2.2)	1.2 (1.6)
Mean number of appointments, *N* (SD)	4.7 (7.3)	3.6 (6.6)	3.2 (4.6)	3.1 (4.8)	2.9 (4.2)	10.5 (12.9)	6.4 (8.5)
Completed treatment, *N* (%)	52,655 (48.0%)	4206 (40.3%)	7236 (46.9%)	5664 (53.7%)	10,459 (71.1%)	3606 (56.7%)	10,348 (42.4%)

Abbreviations: IMD, index of multiple deprivation; N, number of patients; SD, standard deviation.

### 3.2 Age

Age was relatively equally distributed amongst the total adult study population; proportions ranged from 12.2% of patients aged 55–64 years, to 15.1% of patients aged 65 years or older. Approximately 20% of all included patients were pediatric patients (*n*: 21,748, 19.8%; Table 1). Age at first attendance to orthodontics specifically was left skewed, indicating a higher proportion of younger patients. Age at first attendance to prosthetics and oral medicine clinics was right‐skewed, demonstrating a higher attendance for patients aged 55 years and older (Table 1).

### 3.3 Sex

Generally, more females attended LDI (*n*: 58,964 patients, 53.7%) than males (*n*: 50,742 patients, 46.2%). Sex information was missing in 12 of 109,718 recorded patients. Attendances by females were higher than males across most clinics, including hygiene and therapy, prosthetics, periodontal/restorative, orthodontics, oral surgery, and oral medicine. In contrast, more males attended acute dental care (*n*: 6166 patients, 59.0%) than females (*n*: 4280 patients, 41.0%; Table 1).

### 3.4 Deprivation

There were more patients from the most deprived quintile areas (*n*: 43,828 patients, 39.9%) than the least deprived areas (*n*: 9045, 8.2%). Acute dental care clinics had the higher proportion of patients from deprived backgrounds (*n*: 5029 patients, 48.1%; Table 1).

### 3.5 Comorbidities

**Figure 2:**
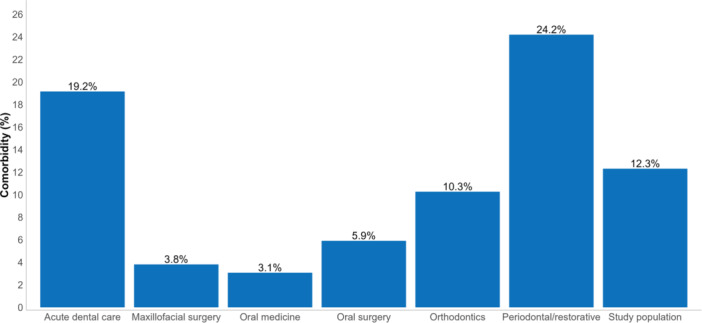
Distribution of any self‐reported patient conditions in the total study population, and by first clinic attended.

**Figure 3:**
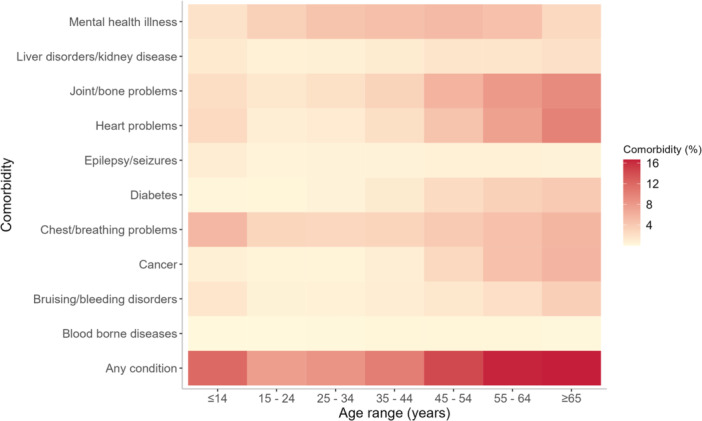
Heat map showing percentage of comorbidities stratified by age group.

Over 10% of the total study population reported having at least one additional comorbidity (12.3%, Figure 2). The most frequently reported comorbidities were joint or bone problems (*n*: 4913, 4.5%), chest or breathing problems (*n*: 4619, 4.2%), and heart problems (*n*: 4455, 4.1%; Table 2). There were higher proportions of patients with comorbidities in older age groups (Figure 3). The prevalence of diabetes was greatest in patients who attended acute dental care (*n*: 355, 3.4%) and periodontal/restorative clinics (*n*: 906, 3.7%; Table 2). In periodontal/restorative clinics, prevalence of comorbidities was greatest with over 20% of patients reporting at least one comorbidity (24.2%; Figure 2) with mental health illness (*n*: 1856, 7.6%), joint or bone problems (n: 2456, 10.1%) and heart problems (*n*: 2043, 8.4%) the most frequently reported comorbidities for this clinic (Table 2).

**Table 2:** Self‐reported conditions in total study population and by first clinic attended.

		Clinic					
	Study population	Acute dental care	Maxillofacial surgery	Oral medicine	Oral surgery	Orthodontics	Periodontal/restorative
Total, *N*	109,718	10,447	15,426	10,539	14,702	6364	24,394
Condition, *N* (%)							
Any	13,497 (12.3%)	2001 (19.2%)	588 (3.8%)	324 (3.1%)	868 (5.9%)	653 (10.3%)	5902 (24.2%)
Blood‐borne diseases	184 (0.2%)	61 (0.6%)	8 (0.1%)	5 (0.0%)	11 (0.1%)	4 (0.1%)	74 (0.3%)
Bruising/bleeding disorders	1715 (1.6%)	267 (2.6%)	90 (0.6%)	46 (0.4%)	120 (0.8%)	41 (0.6%)	686 (2.8%)
Cancer	2384 (2.2%)	236 (2.3%)	207 (1.3%)	83 (0.8%)	225 (1.5%)	23 (0.4%)	1301 (5.3%)
Chest or breathing problems	4619 (4.2%)	635 (6.1%)	209 (1.4%)	111 (1.1%)	280 (1.9%)	292 (4.6%)	1773 (7.3%)
Diabetes	1776 (1.6%)	355 (3.4%)	97 (0.6%)	72 (0.7%)	107 (0.7%)	22 (0.3%)	906 (3.7%)
Epilepsy/seizures	642 (0.6%)	96 (0.9%)	29 (0.2%)	8 (0.1%)	41 (0.3%)	30 (0.5%)	214 (0.9%)
Heart problems	4455 (4.1%)	770 (7.4%)	205 (1.3%)	134 (1.3%)	314 (2.1%)	116 (1.8%)	2043 (8.4%)
Joint or bone problems	4913 (4.5%)	653 (6.3%)	262 (1.7%)	180 (1.7%)	377 (2.6%)	137 (2.2%)	2456 (10.1%)
Liver disorders or kidney disease	1406 (1.3%)	251 (2.4%)	65 (0.4%)	38 (0.4%)	94 (0.6%)	45 (0.7%)	556 (2.3%)
Mental health illness	4103 (3.7%)	726 (6.9%)	207 (1.3%)	110 (1.0%)	331 (2.3%)	188 (3.0%)	1856 (7.6%)
Cardiometabolic	926 (0.8%)	198 (1.9%)	51 (0.3%)	43 (0.4%)	66 (0.4%)	10 (0.2%)	458 (1.9%)

*Note:* Cardiometabolic conditions refer to patients who reported both diabetes and heart problems.

Abbreviations: IMD, index of multiple deprivation; N, number of patients; SD, standard deviation.

### 3.6 Completion of Treatment

For all included patients, the time between the first appointment and final attendance was approximately 1 year (mean: 1.1 years, SD: 1.7); the mean number of appointments per patient was 4.7 (SD: 7.3). On average, patients attended more orthodontic appointments (mean: 10.5, SD: 12.9) and periodontal/restorative clinics (mean: 6.4, SD: 8.5). Approximately 50% of patients had completed their treatment at LDI during the study period (*n*: 52,655 patients, 48.0%). However, this was higher in patients specifically attending oral surgery clinics (*n*: 10,459, 71.1%). Treatment completion was highest in patients aged 14 years or younger, and this was highest in patients who attended oral surgery clinics (79%; Table 1).

## 4 Discussion

This study investigated the common characteristics and completion of dental treatment in patients attending a dental hospital, using data available in their dEHR. Most patients who attended LDI for secondary care dental treatment were female and from more deprived groups. On average, patients were seen at periodontal/restorative and orthodontic clinic appointments more frequently than other clinics. Younger patients and those who attended oral surgery had higher rates of completed treatment. Heart problems, chest or breathing problems, and joint or bone problems were the most recorded comorbidities in the dEHR, while patients who attended periodontal/restorative and acute dental care clinics specifically had the greatest prevalence of systemic disease.

The findings of this study are consistent with previous research assessing sociodemographic trends in dental attendance by sex, age, and deprivation (Camfield et al. 2022). The 2009 Adult Dental Health Survey found that women had higher regular dental attendance rates than men (Chenery 2011). A study in Norway on dental visiting patterns found that women had more frequent dental visits than men and poor finances were also associated with increased dental visits (Nermo and Hadler‐Olsen 2023). Similar findings were made in a study on patients attending a temporomandibular joint disorder clinic based in the United Kingdom (Camfield et al. 2022).

LDI is an NHS provider of secondary care dental services; patients are referred to LDI for free specialist care due to complex needs or requiring urgent dental care. Given that socioeconomic status is an accepted risk factor for poor oral health, and approximately one quarter of the geographic area surrounding Leeds is ranked amongst the most deprived in the United Kingdom (Leeds City Council 2019), it is not surprising that greater proportions of more deprived patients attended LDI (Peres et al. 2019). The findings of this study support the calls for improved equity in access to primary dental care across the United Kingdom (Jo et al. 2021; Newton and White 2021).

The findings also reflect the cumulative risk and the chronic nature of periodontitis and dental caries, and the need for acute dental treatment in emergency situations. Acute dental care was the only clinic to have a higher percentage of males than females. Treatments for acute dental problems are required when a patient experiences sudden onset of pain, swelling, or trauma in the oral cavity. The higher proportion of males attending this clinic could possibly be related to cognitive biases and/or the tendency for men to delay healthcare seeking until the condition becomes intolerable (Shaw et al. 2023). This is a public health concern that affects all healthcare service providers (Wang et al. 2013; Jones et al. 2019).

Comorbidities were highly prevalent, especially in older patients and patients who attended periodontal/restorative clinics. This aligns with previous studies that have demonstrated the association between periodontitis and multimorbidity (Larvin et al. 2021, 2022a). Interestingly, the prevalence of mental health illness was greatest in patients who attended periodontal/restorative clinics. A UK Biobank study found that people with severe mental illness such as psychosis were 40% more likely to have periodontitis (Kang et al. 2023). However, there is conflicting evidence regarding the association between common mental health disorders such as depression/anxiety and periodontitis (Solis et al. 2004; Zheng et al. 2021). These different observed associations could be due to heterogeneity in the study population, or the complex and different stages of the mental health disorders reported in these studies. As such, further epidemiological studies exploring the association between periodontitis and subsequent disease development are required to improve understanding of the wider healthcare burden of periodontitis (Larvin et al. 2022b, 2023). Regular upkeep of comorbidity records within the dEHR, or even linkage to GP/hospital records, will also ensure tailored dental care pathways and more effective treatment plans downstream.

A higher proportion of younger patients, and patients attending oral surgery clinics, completed their treatment. Oral surgery patients are typically referred for urgent or acute procedures, such as tooth extractions or isolated minor oral surgery procedures, which are often delivered within a shorter episode of care. Patients who attend periodontal and restorative clinics are typically referred back to their primary dental care practitioner for ongoing dental treatment and maintenance for their chronic conditions, which could explain the lower secondary care completion rates. Adult patients may also not complete their dental treatment course due to personal reasons, distance from the healthcare provider, or their work schedule (Anagha et al. 2024). A study of primary care dEHR found that pediatric patients are less likely to miss appointments (West et al. 2020); this is also reflected in the higher rate of treatment completion for younger patients in our study population. However, interpretation of treatment completion should take account of the fixed study period. Because the study was restricted to 2014–2023, patients entering the cohort later had shorter follow‐up and therefore less opportunity to complete treatment before the administrative end of the study. This right‐censoring may have led to underestimation of treatment completion among patients seen later in the study period, and temporal comparisons should therefore be interpreted with caution.

To our knowledge, this is the first study to characterize patients attending a dental hospital in the United Kingdom. The dataset is unique in capturing a diverse referral population from Leeds and the surrounding region, although we do not claim that these dEHR data are representative of the general population. The findings should therefore be interpreted in the context of referral bias inherent to secondary and tertiary care: patients referred to LDI are unlikely to represent the wider Leeds population, but may be more appropriately viewed as reflecting patients referred to a UK dental hospital setting. We have described and discussed the characteristics of patients who attended LDI, and a notable strength of this study is the demonstrable utility of dEHR in epidemiological research. While the findings may only represent patients who require specialist secondary dental care, the results can be used to understand the demography of these patients, as well as the healthcare needs of people from more deprived areas. Several limitations should also be acknowledged. Comorbidity information was self‐reported, not independently validated, and may be subject to recall or reporting bias. While analyses on established cohorts such as the National Health and Nutrition Examination Survey utilize similar self‐reported variables (Centers for Disease Control and Prevention CDC 2024), there are concerns about the possibility of inaccuracies and the impact this may have on statistical associations (Sulieman et al. 2022). As this study was descriptive, underreporting of comorbidities may have affected the absolute prevalence estimates, although the relative proportions across clinics and demographics, and overall conclusions are less affected. Finally, because of routine administrative data in dEHR, information on some important variables, such as ethnicity, smoking status, or BMI, was not routinely recorded. This highlights an important lesson for future dEHR‐based studies: linkage requests and data extraction specifications should, where possible, include key demographic, behavioral, and clinical risk factors to support more complete characterization of patients attending secondary dental care.

This study has demonstrated the need for future epidemiological and inferential analyses that will interrogate health inequalities and dissect oral‐systemic associations further, particularly in UK populations. Though the findings may not be generalized to primary care dentistry, clinicians and researchers can use this information to tailor care management plans and design future epidemiological studies, respectively. We have also shown the feasibility for using routinely collected data in epidemiological dental research, using the variables available within dEHR. As comorbidity information was self‐reported, further study into the associations with clinical systemic diagnoses was not possible. Linkage of dEHR to secondary care hospital records such as HES would allow researchers to dissect the complex relationship between oral conditions and systemic health and provide novel research insights. This research could be pivotal for incorporating oral disease management pathways in chronic disease preventive initiatives and reducing overall healthcare burden.

## 5 Conclusion

This study revealed greater proportions of patients seen at Leeds Dental Hospital were female and from more deprived groups. Frequently reported conditions included heart, chest, breathing, joint or bone problems. Patients attending periodontal/restorative clinics had the greatest prevalence of comorbidities. Future linkages of dEHR with hospital records could further improve understanding of the complexities of oral‐systemic associations.

## Author Contributions

Jing Kang and Jianhua Wu conceived and designed the study, performed statistical analysis, interpreted results, and contributed to the drafting and critical revision of the manuscript. Chenyi Gao analyzed the results and contributed to the drafting and critical revision of the manuscript. Sue Pavitt conceived and designed the study, interpreted results, and contributed to the drafting and critical revision of the manuscript. Nikolaos Donos and Vanessa Muirhead provided clinical interpretation of results and contributed to the drafting and critical revision of the manuscript.

## Ethics Statement

This project has obtained HRA and Health and Care Research Wales Approval (IRAS project ID 277767, REC reference [Shaw et al. 2023]/WM/0127, Sponsor: University of Leeds).

## Conflicts of Interest

The authors declare no conflicts of interest.

## Supporting information


Supporting File

## Data Availability

The data that support the findings of this study are available on request from the corresponding author. The data are not publicly available due to privacy or ethical restrictions.
